# *Peromyscus* spp. Deer Mice as Rodent Model of Acute Leptospirosis

**DOI:** 10.3201/eid3107.241579

**Published:** 2025-07

**Authors:** Ellie J. Putz, Claire B. Andreasen, Paola Boggiatto, Mitchell V. Palmer, Luis G.V. Fernandes, Bienvenido W. Tibbs-Cortes, Judith A. Stasko, Camila Hamond, Steven C. Olsen, Jarlath E. Nally

**Affiliations:** National Animal Disease Center, US Department of Agriculture Agricultural Research Service, Ames, Iowa, USA (E.J. Putz, P. Boggiatto, M.V. Palmer, L.G.V. Fernandes, B.W. Tibbs-Cortes, J.A. Stasko, S.C. Olsen, J.E. Nally); Iowa State University College of Veterinary Medicine, Ames (C.B. Andreasen); National Veterinary Services Laboratories, US Department of Agriculture Animal and Plant Health Inspection Service, Ames (C. Hamond)

**Keywords:** leptospirosis, zoonoses, bacteria, *Leptospira interrogans*, *Leptospira borgpetersenii*, foam cells, foamy macrophages, macrophage, Golden Syrian Hamster, *Peromyscus leucopus*, deer mouse, sex effect, United States

## Abstract

Leptospirosis is a global zoonotic disease affecting humans, wildlife, companion, and domestic animals. Incidental hosts can contract the disease directly or indirectly from asymptomatic reservoir hosts, most commonly small rodents. The Golden Syrian hamster is recognized as the dominant rodent model for acute leptospirosis because the animals are susceptible to many serovars and are used to maintain laboratory strains and test bacterin vaccine efficacy. However, hamsters are primarily used in survival-based studies, and investigations into host immune response and disease pathogenesis are limited. We found that *Peromyscus leucopus* white-footed deer mice are susceptible to acute leptospirosis, and thus might be an alternative rodent model. Furthermore, similar to hamsters, deer mice produce circulating foamy macrophages in response to *Leptospira* challenge. Deer mice exhibit differences in response to different serovars, clinical disease severity, kidney and liver lesions, and an overall sex effect, with male mice demonstrating more severe clinical signs and higher bacterial burden.

Leptospirosis is a global zoonosis causing severe disease in humans, domestic animals, and wildlife (*1*). In cattle and most livestock species, leptospirosis causes abortions and reproductive losses, resulting in serious veterinary health and economic concerns for producers (*2*,*3*). Commercial vaccines are available but are not cross-protective across *Leptospira* serogroups. Because of the range in clinical sign severity and the fastidious nature of culturing the pathogen, leptospirosis diagnosis can be challenging, and the disease is consistently underrecognized by clinicians. *Leptospira* spp. bacteria colonize the kidneys and are typically shed by reservoir hosts in their urine, where they are transmitted from the environment to incidental hosts (*4*). The primary reservoir hosts are members of the rodent family. Infected mice and rats are largely asymptomatic, and cohabitation in field environments, barns, and dwellings presents ample opportunity to foster transmission to humans and domestic animals.

The small animal models used to study acute leptospirosis are hamsters, guinea pigs, and gerbils (*5*–*7*). Although vaccines exist for many domestic species, commercial bacterins require validation before release. For those purposes, Golden Syrian hamsters (*Mesocricetus auratus*) are widely considered the standard model for leptospirosis research (*8*). Unlike most wildlife rodents, hamsters demonstrate acute and typically fatal signs of infection when challenged, although severity of disease can vary among *Leptospira* serovars, species, and strains (*9*–*11*). Hamsters are used to passage laboratory strains for research purposes, such as tests or the recovery of virulence (*8*). Similarly, hamsters are used in survival studies to test commercial bacterin vaccines (as required by the US Department of Agriculture), regardless of the intended host species (*12*). Although the research community relies heavily on the hamster model, it has notable limitations. They are mostly used for survival metrics instead of disease mechanism or immunology–based studies and suffer from a lack of reagents (such as markers of immune cell types) and resources (such as genomic/proteomic databases). Further, hamsters are largely not found in the wild and thus do not accurately model naturally occurring leptospirosis transmission.

*Peromyscus* mice, commonly known as deer mice, are phylogenetically more similar to hamsters in the Cricetidae family than are the classic Muridae *Mus musculus* mice (*13*,*14*). Wild deer mice, including a variety of subspecies (one of the most abundant being *P. leucopus* white-footed deer mice), are found in parts of North America and are used for bacterial and viral disease research, including research into Lyme disease, anaplasmosis, hantavirus (*P. maniculatus* mice), and viral encephalitis (*15*,*16*). For many of those diseases, deer mice serve as reservoir hosts and play crucial roles in tickborne disease life cycles and transmission kinetics, where they are characterized by a lack of clinical signs and reduced inflammatory immunological responses (*16*,*17*). Some aspects of the transmission and movement of strains of *Leptospira* from wildlife to livestock are unknown, and deer mice might serve as a potential environmental wildlife reservoir similar to other documented rodent hosts (*18*).

Our group strives to improve tools and metrics to assess immunological responses in rodent models that are key for leptospirosis research. Previously, we established that hamsters produce circulating foamy macrophages in response to *Leptospira* and bacillus Calmette-Guérin challenge, and the presence of those cell populations is correlated with disease severity (*11*,*19*,*20*). Foamy macrophages are best known for their characteristic appearance in the tissue of granulomas caused by *Mycobacterium tuberculosis* complex but are not typically found in circulation. Foamy macrophage vacuoles contain defined lipid droplets, but unlike the foamy macrophages that contain mycobacteria in tuberculous granulomas, those cells do not appear to contain leptospires in the challenged hamster model (*11*,*21*). In this study, we sought to assess the suitability of *Peromyscus* spp. mice as an alternative rodent model of leptospirosis. 

## Materials and Methods

### Animals

We obtained *P. leucopus* deer mice 6–13 weeks of age from the LL stock *Peromyscus* Genetic Stock Center at the University of South Carolina (Columbia, SC, USA). All procedures and experiments were approved by the National Animal Disease Center Animal Care and Use Committee. Deer mice were monitored daily for health evaluation and had ad libitum access to food and water.

### Experimental Design

This work includes data from 3 independent experiments. *Leptospira borgpetersenii* serogroup Ballum serovar Arborea strain LR131 and *L. interrogans* serogroup Canicola serovar Canicola strain LAD1 were propagated at 29°C in HAN media (*22*). Animals were challenged by intraperitoneal injection with 0.5 mL containing 1 × 10^7^ leptospires (LR131 or LAD1) or 0.5 mL HAN media alone. Noninjection (negative) male and female control mice were used for nontreatment comparisons.

We monitored deer mice daily for clinical signs including lethargy; blood in urine; blood from nose, urogenital tract, or on paws; or lack of grooming/hygiene. We euthanized animals at the appearance of severe clinical signs such as blood from nose, paws, or urogenital tract or extreme lethargy (monitored by response to a human handler). We collected whole blood smears, kidney and liver samples for culture and quantitative PCR (qPCR) and tissues for histopathologic examination (spleen, lung, heart, kidney, liver, pancreas, and omental adipose).

Because severe clinical signs developed in male and female mice at different timepoints, we harvested control animals of the opposite sex as necessary. In a subset of female mice challenged with *L. interrogans* strain LAD1, acute signs of disease did not develop; we euthanized those mice ≈3 weeks postchallenge.

### Culture and qPCR

For all challenged animals, we harvested a kidney or section of liver and macerated the specimen in 5 mL of HAN media containing 5-fluorouracil (100 µg/µL) for *Leptospira* culture. We used numerous dilutions of macerated suspensions to inoculate HAN media plus 5-fluorouracil as described previously (*22*) and incubated at 37°C in 5% CO_2_ (*23*). We determined bacterial burden of liver and kidney by using genomic *lipL32* qPCR as previously reported (*19*).

### Blood and Tissue Processing and Evaluation

We collected blood smear slides using the feathered edge technique, stained them with Giemsa solution, and evaluated them by microscopy. We performed a 100-cell differential count classifying the number of neutrophils, lymphocytes, monocytes, foamy macrophages, and combined eosinophils and basophils.

We took tissue samples of similar size and section for all groups from liver, kidney, spleen, lung, heart, and omental adipose tissue. We collected tissues into formalin and transferred to 70% ethanol after 24 hours. We used standard paraffin-embedding techniques to further process the fixed samples. We transferred cut paraffin-embedded tissues sections (4-μm-thick sections) to Superfrost Plus charged microscope slides (Thermo Fisher Scientific, https://www.thermofisher.com), after which we stained them with hematoxylin and eosin. We evaluated histologic sections by light microscopy using an Olympus B41 microscope and DP 23 Olympus camera and captured images with cellSens Olympus software (Evident Scientific, https://evidentscientific.com).

We performed immunohistochemistry with the Ventana Ultra Discovery on representative formalin-fixed, paraffin-embedded tissue sections 4 μm thick. We deparaffinized sections in Discovery Wash (Roche, https://www.roche.com) and achieved antigen retrieval by incubation with cell-conditioning solution 1, a citrate-based buffer (pH 6.0). We performed a blocking step with Discovery Goat Ig Block (Roche) for 20 minutes at 35°C. We incubated sections for 32 minutes at 35°C with primary antibody against LipL32 at 1:100 dilution in Discovery antibody diluent (Roche). The signal was detected with rabbit Dako EnVision-HRP system (Agilent, https://www.agilent.com) for 32 minutes at 35°C and visualized with 3,3′-diaminobenzidine (Roche). We counterstained the sections with Harris Hematoxylin (Leica Biosystems, https://www.leicabiosystems.com).

### Statistics

We evaluated stained whole-blood smear cell counts (cell numbers/100 leukocytes) using simple linear regression models in R version 4.1.0 (The R Project for Statistical Computing, https://www.r-project.org). We evaluated each cell type independently (lymphocytes, neutrophils, monocytes, foamy macrophages, and combined eosinophils and basophils). For bacterial burden data, we evaluated genomic *lipL32* qPCR independently between kidney and liver. For all, each group was fit as a fixed effect detailing male mice with acute disease, female mice with acute disease, and time-matched nonclinical control female mice (showing no signs of disease) taken when acute male mice reached endpoint. We included 2 control groups, media alone and noninjection negative controls. We evaluated specific contrasts within cell types with pairwise contrasts. We determined significance by a p value of <0.05; error bars represent SEs. We analyzed Spearman correlations in R between foamy macrophage counts and bacterial burden of liver and kidney across all deer mice.

## Results

**Figure 1:**
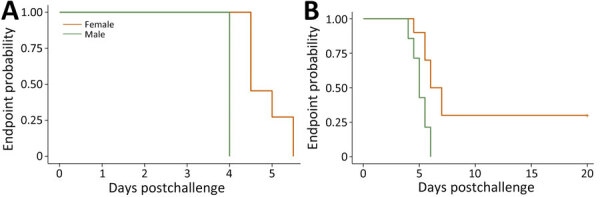
Endpoint and survival curves for *Peromyscus* deer mice experimentally infected with *Leptospira* in study of the species as rodent model of acute leptospirosis. Graphs indicate survival curves for severely diseased female and male deer mice infected with *Leptospira borgpetersenii* strain LR131 (A) and *L. interrogans* strain LAD1 (B). p value <0.01 between sexes for both LR131- and LAD1-infected animals.

We challenged acclimated male and female *Peromyscus* spp. deer mice with *L. interrogans* serogroup Canicola serovar Canicola (strain LAD1) and *L. borgpetersenii* serogroup Ballum serovar Arborea (strain LR131) and evaluated both media alone and noninjection controls. Deer mice showed severe clinical signs of disease within the first week postchallenge, establishing them as an alternative rodent model of acute leptospirosis (Figure 1). Although both *L. borgpetersenii* and *L. interrogans* strains caused acute disease, endpoint separation between male and female mice is striking and demonstrates that male mice were more susceptible than female mice to both LR131 and LAD1 (p<0.01 between sexes for both) (Figure 1). Because male mice were more susceptible, we also evaluated a control group of nonclinical female mice not displaying severe signs of disease but euthanized at the same time as acutely ill male mice.

**Figure 2:**
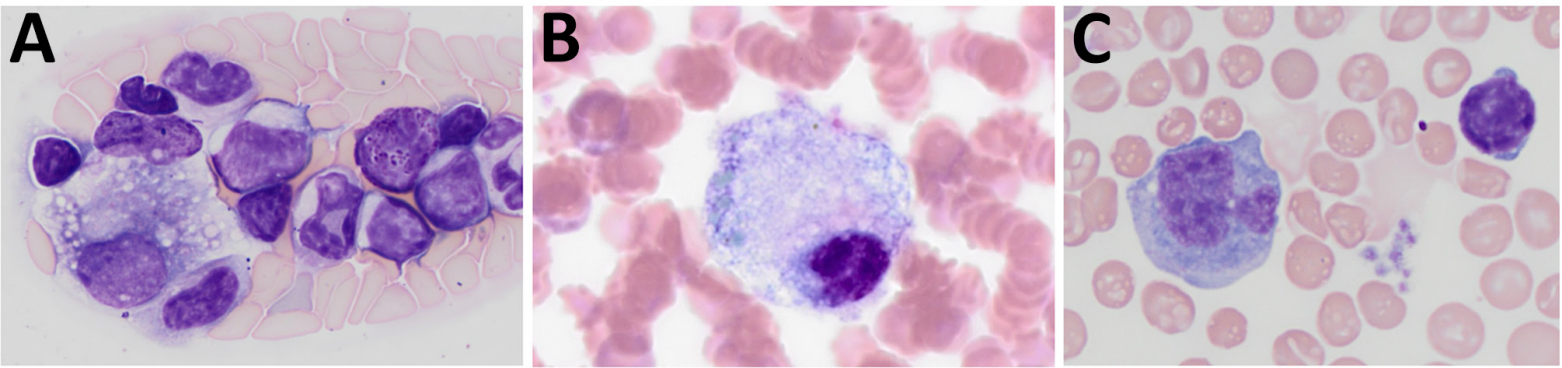
Whole blood smears depicting foamy macrophages in study of *Peromyscus* species deer mice as rodent model of acute leptospirosis. Representative blood smears containing foamy macrophages mice experimentally infected with *Leptospira borgpetersenii* strain LR131 (A), *L. interrogans* strain LAD1 (B), and noninfected control monocyte (C) are shown. Original magnification ×100.

**Figure 3:**
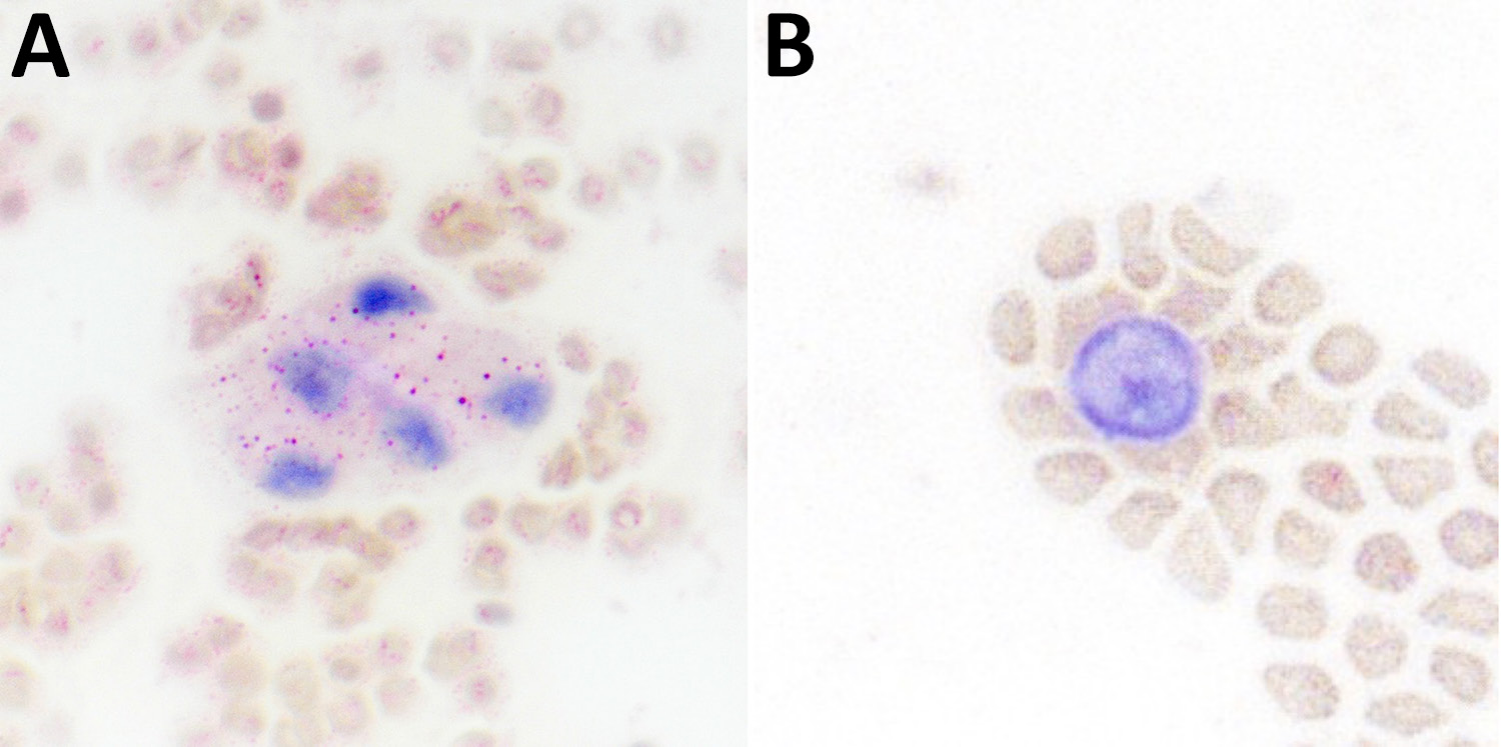
Microscopy presentation of lipid within foamy macrophages from infected mice in study of *Peromyscus* species deer mice as rodent model of acute leptospirosis. Representative Oil Red O-stained whole blood smears from a *Leptospira* infected animal (A) and a noninfected control (B) are shown. Original magnification ×20.

**Figure 4:**
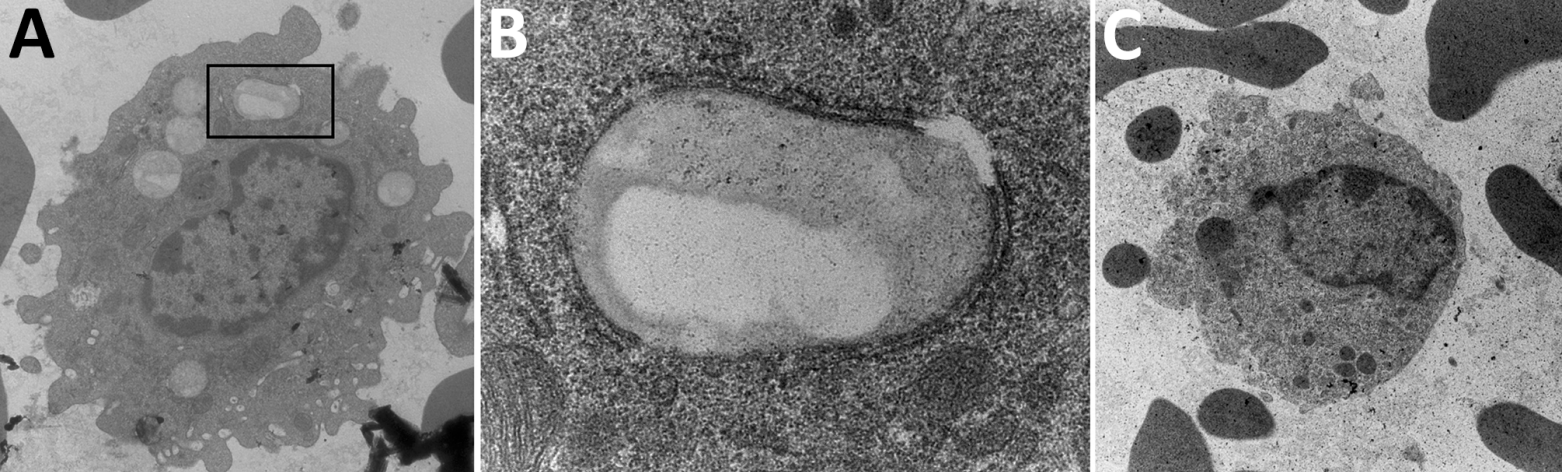
Transmission electron microscopy presentation of whole blood indicating foamy macrophages from infected mice in study of *Peromyscus* species deer mice as rodent model of acute leptospirosis. A) Image of foamy macrophages with membrane-bound lipid droplet in black box; original magnification ×6,800. B) Close-up of membrane-bound lipid droplet from box in panel A (original magnification ×49,000). C) Noninfected control monocyte (original magnification ×6,800).

Previously, our group described the unique production of circulating foamy macrophages by hamsters challenged with *Leptospira* (*11*), a feature not replicated in experimentally infected laboratory mice, guinea pigs, or rats (E. Putz et al., unpub. data). By examining Giemsa-stained slides of whole blood smears, we identified circulating foamy macrophages, consistent with those described in hamsters, in deer mice (Table; Figure 2). Intracellular lipid droplets are defining features of foamy macrophages. We confirmed the presence of those lipids with microscopy techniques including Oil Red O staining (Figure 3) and transmission electron microscopy (Figure 4). Similar to findings in the hamster model, no leptospires were detectable within foamy macrophages by transmission electron microscopy (*24*). Additional hematologic findings included neutrophilia and lymphopenia (Table).

**Table:** Manual differential cell counts on Giemsa-stained whole blood smears in study of *Peromyscus* species deer mice as rodent model of acute leptospirosis*

Group	Cells/100 leukocytes				
	Foamy macrophages	Monocytes	Neutrophils	Lymphocytes	Eosinophils and basophils
LR131 female	3.75 + 1.25†	3.08 + 0.54†	34.00 + 3.12†	58.2 + 3.40†	0.92 + 0.48
LR131 male	7.63 + 1.53†	4.13 + 0.66†	36.62 + 3.82†	50.4 + 4.17†	0.88 + 0.59
LR131 nonclinical female	0.33 + 2.50	1.33 + 1.07	47.33 + 6.24†	49.7 + 6.81†	1.33 + 0.96
LAD1 female	1.70 + 1.37	2.00 + 0.59	24.00 + 3.42	70.6 + 3.73†	1.70 + 0.53
LAD1 male	3.36 + 1.16†	2.86 + 0.50†	35.50 + 2.89†	55.1 + 3.15†	3.14 + 0.45†
LAD1 nonclinical female	0.50 + 2.17	1.75 + 0.93	26.50 + 5.41	68.2 + 5.90†	2.75 + 0.84†
HAN media	0.22 + 0.90	1.04 + 0.39	16.26 + 2.26	81.6 + 2.46	0.87 + 0.35
Negative	0.00 + 1.53	0.88 + 0.66	8.38 + 3.82	90.6 + 4.17	0.13 + 0.59

*Table shows the least square means of foamy macrophages, monocytes, neutrophils, lymphocytes, and combined eosinophils and basophils, with their respective SEs. †Denotes a significant difference (p<0.05) compared with HAN media controls.

**Figure 5:**
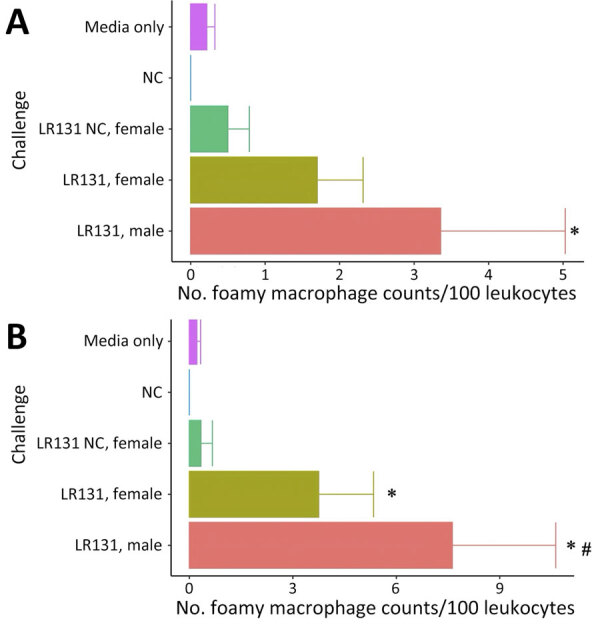
Concentrations of foamy macrophages per 100 leukocytes in whole blood smears from male and female *Peromyscus* deer mice in study of the species as rodent model of acute leptospirosis. Concentrations of foamy macrophages are shown for acute disease male, acute disease female, and NC female mice taken when acute male mice reached endpoint for challenge with *Leptospira interrogans* strain LAD1 (A) or *L. borgpetersenii* strain LR131 (B). Error bars depict SEs. Asterisk denotes a significant difference (p<0.05) from media alone and negative control groups, whereas pound sign denotes a significant difference (p<0.05) between male and female groups. NC, nonclinical.

Substantial populations of foamy macrophages were found in the blood of deer mice challenged with both *L. borgpetersenii* and *L. interrogans* (Figure 5). Control noninjected animals did not produce any foamy macrophages, but media alone controls had sparse production of an occasional foamy-filled cell, suggesting a possible immune response to HAN media. LAD1-challenged male mice produced significantly more foamy macrophages than did media alone (p<0.01) and negative control (p = 0.02) animals. Male LAD1-infected deer mice also produced more foamy macrophages (3.36 + 1.16/100 counted leukocytes) than did acute female (1.70 + 1.37/100 counted leukocytes; p = 0.22) or control female counterparts (0.50 + 2.17/100 counted leukocytes; p = 0.12), but differences were not significant (Figure 5, panel A). Differences among challenged groups were stronger for *L. borgpetersenii* LR131–infected animals, whereas male deer mice generated significantly greater numbers of foamy macrophages than did acute female (p = 0.05), nonclinical control female (p = 0.01), HAN media alone (p<0.01), or negative control (p<0.01) groups (Figure 5, panel B). Given that LR131-challenged deer mice reached endpoints more quickly than LAD1-challenged deer mice, foamy macrophage cell percentages appear to be associated with disease severity. This finding further corroborates the sex effect seen in this study; higher foamy macrophage proportions were found in male mice than in acutely diseased female mice (Figure 5).

No significant histologic findings were present in the HAN media only and negative control groups. Challenge groups for strain LR131 and strain LAD1 had variable and nonspecific changes in the spleen and lung. The spleen had variable lymphoid follicle expansion, which can result from antigenic stimulation, and extramedullary hematopoiesis. The lung had variable hemorrhage and congestion; some vessels contained neutrophils, attributed to the peripheral blood neutrophilia.

**Figure 6:**
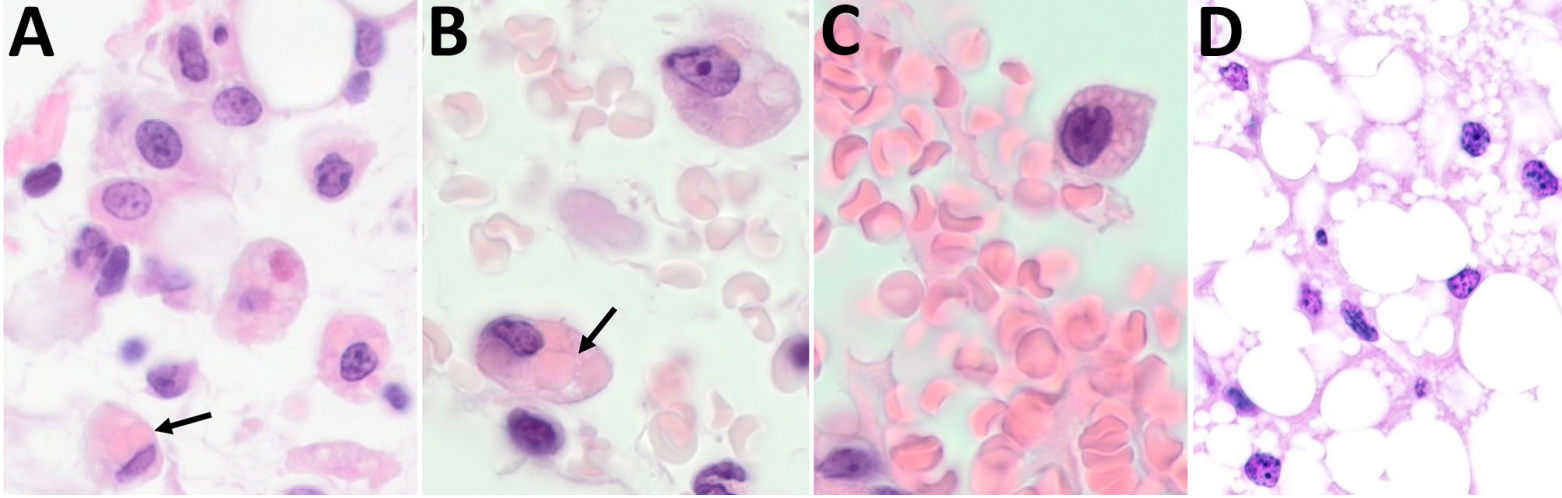
Histological examination of omental tissue from *Peromyscus* deer mice in study of the species as rodent model of acute leptospirosis. A) *Leptospira borgpetersenii* strain LR131–challenged deer mouse (original magnification ×40); B) foamy macrophages with intracytoplasmic erythrocytes and clear lipid vacuoles in sample taken from LR131-challenged deer mouse (original magnification ×100); C) foamy macrophage within vessel lumen and foamy macrophage, also present in peripheral blood *L. interrogans* strain LAD1–challenged deer mouse (original magnification ×100); D) omentum sample from noninfected deer mouse (original magnification ×40). Black arrows indicate possible foamy macrophages/macrophages with intracytoplasmic erythrocytes (erythrophagocytosis).

Both strains LR131 and LAD1 kidney-associated omental adipose tissue had hemorrhage and inflammatory cell infiltrates when associated with kidney inflammatory lesions. Cell infiltrates consisted of variable numbers of neutrophils, lymphocytes, macrophages, and suspected foamy macrophages (Figure 6). Some macrophages, or possible foamy macrophages, contained erythrocytes (Figure 6), also seen in the blood (Figure 2). Immunohistochemistry also confirmed the presence of leptospires within kidney adipose tissue (Appendix Figure).

**Figure 7:**
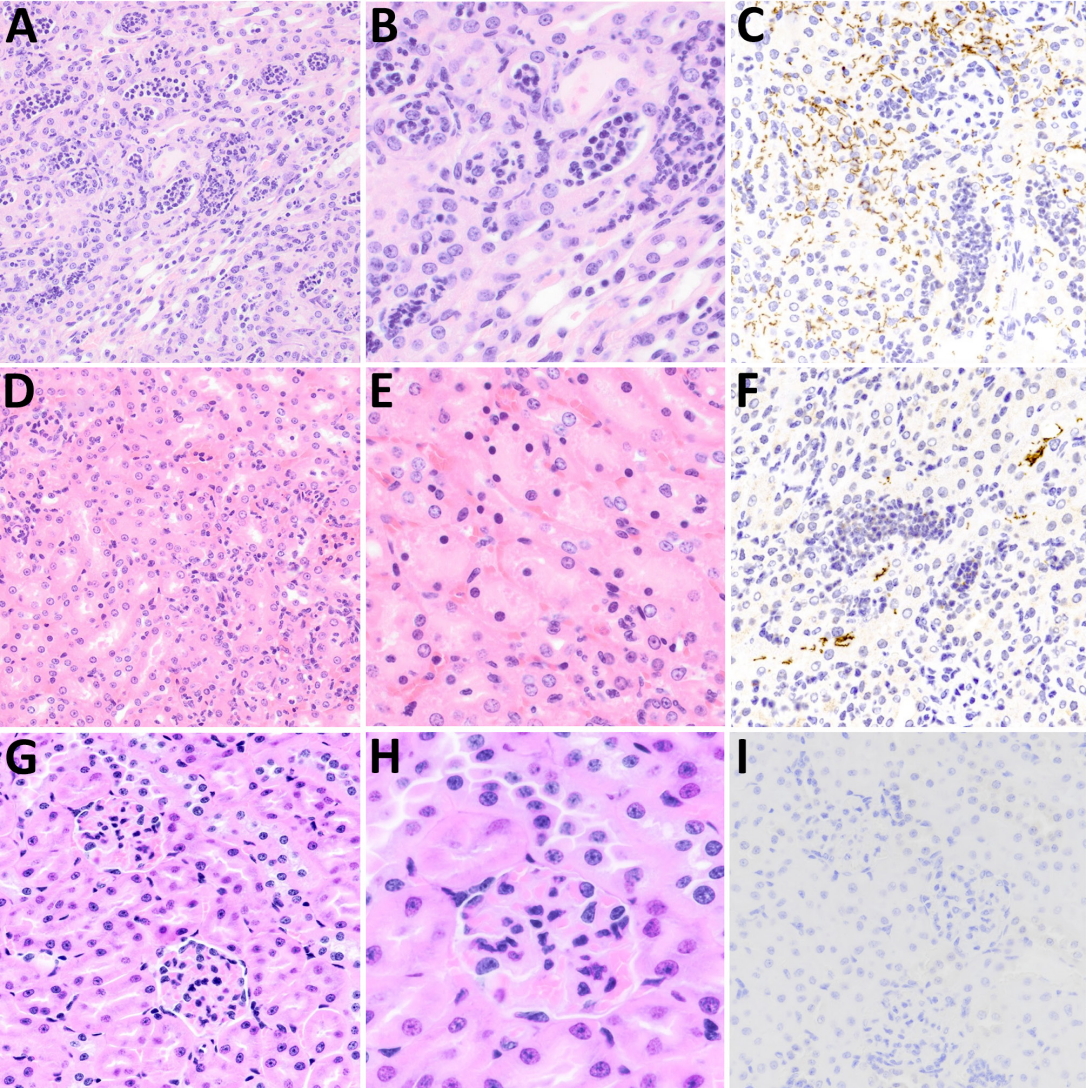
Microscopic and immunohistochemical examination of kidney samples taken from challenged and control deer mice in study of *Peromyscus* species deer mice as rodent model of acute leptospirosis. A–C) Samples taken from LR131-challenged deer mice showing acute diffuse purulent nephritis and nephrosis. The renal cortex and medulla appeared similar with severe diffuse intratubular neutrophilic inflammation, loss and necrosis of tubular lining cells, and resulting nephrosis. Intertubular neutrophilic infiltrates in the interstitium and some neutrophilic glomerular infiltrates (not common) were present. There were scattered areas of hemorrhage (not pictured). A, B) Hematoxylin and eosin–stained tissue (A, original magnification ×20; B, original magnification ×40); C) LipL32 immunohistochemistry-labeled kidney (original magnification ×20). D–F) Samples taken from LAD1-challenged deer mice showing most severe renal lesion associated with LAD1. Examination showed acute multifocal purulent nephritis and nephrosis with lymphocytic and slightly purulent interstitial nephritis. The renal cortex contained multifocal intratubular neutrophilic inflammation, loss and necrosis of tubular lining cells with the accumulation of eosinophilic material and resulting nephrosis. Intertubular lymphocytes and fewer neutrophils were scattered in the interstitium. D, E) Hematoxylin and eosin–stained tissue (D, original magnification ×20; E, original magnification ×40); F) LipL32 immunohistochemistry-labeled kidney (original magnification ×20). G–I) Sample of noninfected control animal kidney. G) Hematoxylin and eosin–stained tissue (G, original magnification ×20; H, original magnification ×40); I) LipL32 immunohistochemistry negative (original magnification ×20).

Significant lesions of interest were present in the kidney and liver and varied between challenge strains; inflammatory lesions in the kidney were more severe and diffuse in a higher number of deer mice challenged with strain LR131 than those challenged with LAD1 (Figure 7). The LR131-challenged acute disease group had renal cortex intratubular neutrophils that extended into the medulla, tubular necrosis, interstitial neutrophilic nephritis, hemorrhage, and perivascular neutrophils and lymphocytes (Figure 7). The LAD1-challenged acute disease group had similar renal lesions, but they were less severe and diffuse, and hemorrhage and medullary inflammatory infiltrates were rare. Lesion severity differences were further supported by immunohistochemistry, which demonstrated a greater number of leptospires in LR131-infected mice (Figure 7).

**Figure 8:**
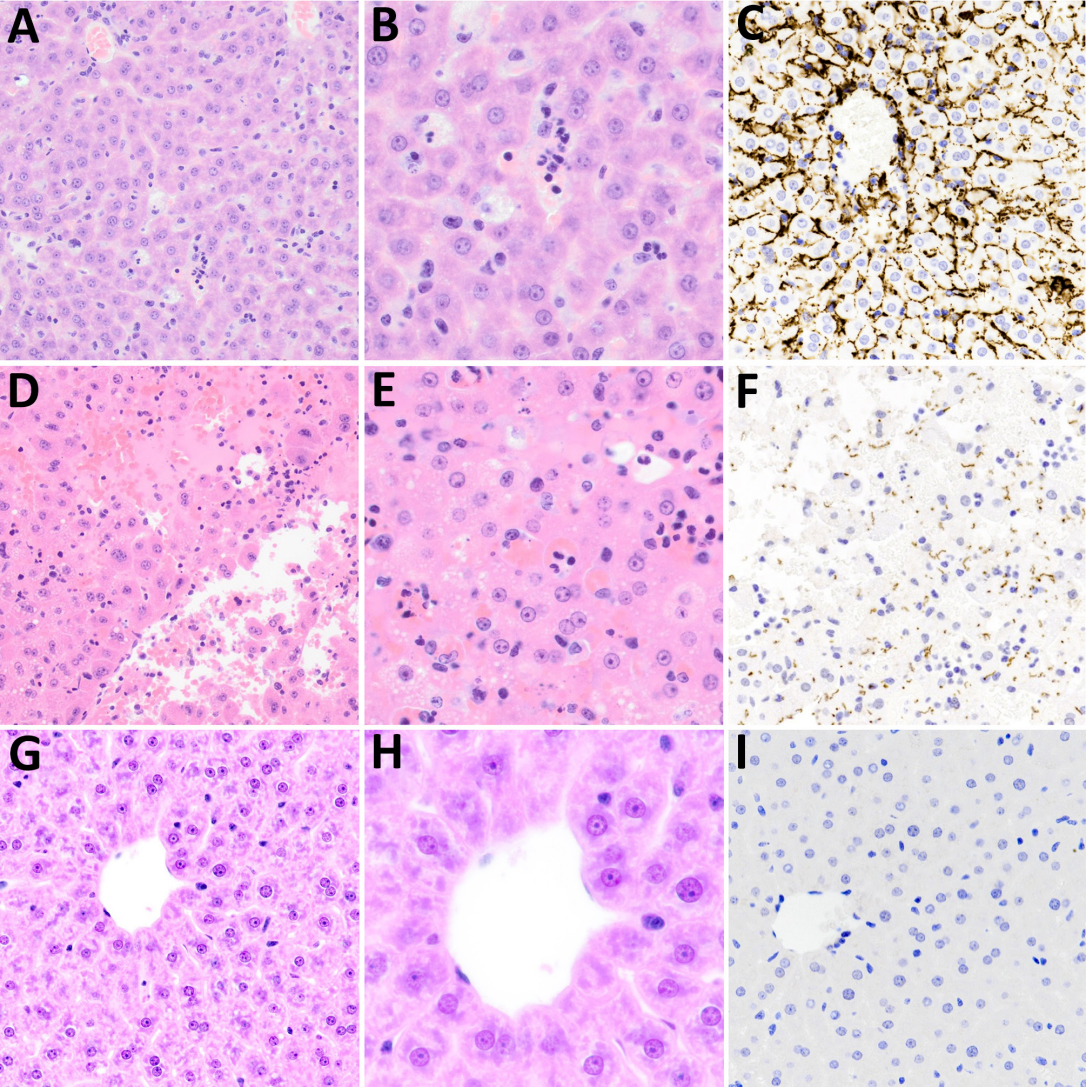
Microscopic and immunohistochemical examination of liver samples from challenged and control deer mice in study of *Peromyscus* species deer mice as rodent model of acute leptospirosis. A–C) Samples from LR131-challenged deer mice showing minimal change and intravascular neutrophils. The liver had intact architecture, minimal hepatocyte change, and some areas of vascular intrasinusoidal neutrophils. The presence of neutrophils may represent peripheral blood versus recruitment to the liver. A) Hematoxylin and eosin–stained tissue (A, original magnification ×20; B, original magnification ×40); C) LipL32 immunohistochemistry-labeled liver (original magnification ×20). D–F) Samples from LAD1-challenged deer mice showing acute diffuse purulent hepatitis and necrosis. There was marked disruption of hepatic architecture with hepatocyte dissociation, diffuse neutrophilic (purulent) inflammation, and hepatocyte necrosis. Necrosis of hepatocytes resulted in confluent midzonal and centrilobular necrosis, sometimes bridging, with hemorrhage. The necrosis resulted in tissue fragility. D) Hematoxylin and eosin–stained tissue (A, original magnification ×20; B, original magnification ×40); F) LipL32 immunohistochemistry-labeled liver (original magnification ×20). G–I) Sample of noninfected control animal liver. G) Hematoxylin and eosin–stained tissue (A, original magnification ×20; B, original magnification ×40); I) LipL32 immunohistochemistry negative (original magnification ×20).

In the histologic assessment of the liver, animals infected with strain LR131 had mild hepatic lesions consisting of intrasinusoidal neutrophils, possibly associated with peripheral neutrophils, and low numbers of occasional perivascular lymphocytes (Figure 8). This finding contrasted with the LAD1-challenged group, which had mild to severe diffuse liver lesions (Figure 8). The contrasting severity of lesions and density of leptospires differences were confirmed by immunohistochemistry (Figure 8). Milder lesions consisted of hepatocyte vacuolar change, hepatocyte swelling (cell edema), and a few areas of perivascular lymphocytic inflammation. Severe liver lesions consisted of hepatocyte dissociation and purulent hepatocyte necrosis that was multifocal to diffuse with a midzonal to centrilobular pattern, often bridging (Figure 8).

**Figure 9:**
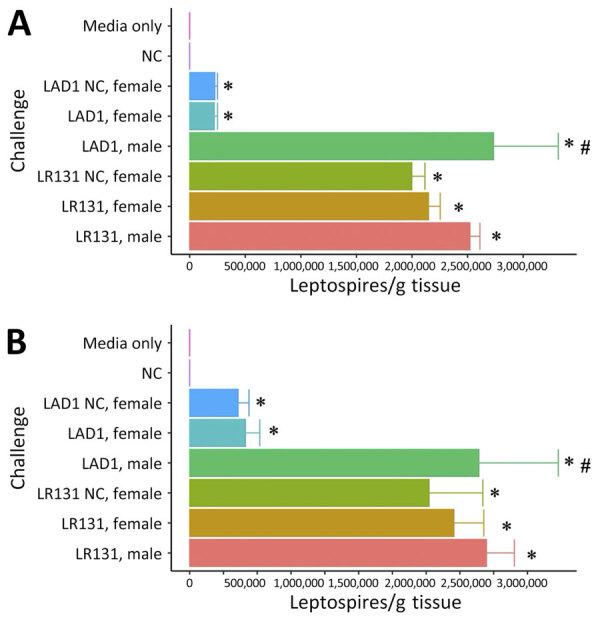
Genomic equivalent burden of calculated leptospires per gram of tissue between male and female *Peromyscus* deer mice in study of the species as rodent model of acute leptospirosis. Graphs show the PCR-calculated bacterial load in infected kidneys (A) and livers (B) from deer mice infected with strain LR131 *Leptospira borgpetersenii* or strain LAD1 *L. interrogans*, HAN media alone, and noninfected negative control. Groups shown consist of acute disease male mice, acute disease female mice, and NC female mice taken when acute male mice reached endpoint. Error bars depict SEs. Asterisk denotes a significant difference (p<0.05) from media alone and negative control groups, whereas pound sign denotes a significant difference (p<0.05) between male and female groups. NC, nonclinical.

Culture was positive in liver and kidney samples from all challenged animals, and we quantified bacterial load by using genomic equivalent qPCR for *lipl32*. Bacterial loads were similar between challenges, but the highest levels of leptospires per gram were seen in the liver (Figure 9). For both *Leptospira* species–infected deer mice, bacterial load was higher in kidney and liver samples from male mice than in samples from acute female, nonclinical female, HAN media alone, and negative control groups (Figure 9, panels A, B) (p<0.01). As seen previously in hamsters (*25*), between LAD1- and LR131-challenged female mice, bacterial burdens were higher in both liver and kidney samples for *L. borgpetersenii* challenge than *L. interrogans* (p<0.01). In contrast, bacterial load in male mice did not differ in kidney (p = 0.56) or liver (p = 0.33) specimens between challenge strains (Figure 9). Foamy macrophage percentages were significantly correlated with bacterial load of leptospires in liver and kidney specimens (p<0.01), further demonstrating their association with virulence.

## Discussion

Acute disease developed in *P. leucopus* deer mice in response to *Leptospira* challenge, offering an alternative acute rodent model to elucidate pathogenic mechanisms of leptospirosis. From an evolutionary standpoint, deer mice are more closely related to hamsters than to *M. musculus* mice (*13*,*14*). In addition to displaying severe clinical signs such as hematuria, blood on nose and foot pads, and lethargy, deer mice produced foamy macrophages in response to both *L. interrogans* and *L. borgpetersenii* challenge, which correlated with disease severity. Our group previously identified that hamsters produce circulating foamy macrophages in response to bacterial infection, but in our studies, those cells do not appear in mouse, guinea pig, or rat models of leptospirosis. Because circulating foamy macrophages are present in low numbers and can be unevenly distributed in blood smears, we found visual examination of the entire blood smear (versus a 100-cell count differential) was sometimes needed to detect foamy macrophages. Also, foamy macrophages can be missed if only automated analyzer evaluation is used for hematology analysis (with specific parameters flagged to require visual examination, as can occur in human medicine). This work suggests that circulating foamy macrophages might be limited to the Cricetidae family, but evaluation of additional rodent species, further investigation in *Peromyscus* subspecies, and review of blood smears from other species susceptible to leptospirosis would be necessary to confirm this unique finding.

*Peromyscus* spp. mice are commonly recognized as asymptomatic reservoir hosts, particularly for the Lyme disease bacteria *Borrelia burgdorferi*. Much work has been done exploring the reservoir host components and pathogen interactions between *Peromyscus* species and other rodents. Gene expression work suggests *Peromyscus* spp. mice respond differently than *M. musculus* mice to lipopolysaccharide challenge (*17*,*26*) and have altered immune cell interactions, such as largely inactive or altered immunoglobulin gamma Fc receptors (*27*). Previous work in deer mice has established that *Peromyscus* transcriptomic profiles are more aligned with M2 macrophage than M1 polarization in response to endotoxin (*26*) and are associated with alternatively activated monocytes (*17*). Specifically, deer mice showed a low inducible nitric oxide synthase 2 (Nos2), high arginase 1 (Arg1) expression ratio in response to lipopolysaccharide challenge (*28*), which is typically associated with a damage repair or antiinflammatory response. Such nonclassical activation profiles could contribute to the production of foamy macrophages, but the mechanism of circulating foamy macrophage formation and their functional capabilities are still unclear. Implications of foamy macrophages for host immune response and bacterial persistence are also of interest, because the acquisition of low-density lipoprotein cholesterol within foamy macrophage vacuoles is viewed as a nutrient source for pathogens (*29*). Of note, foamy macrophages have been tissue-associated with other infectious agents, such as *Chlamydia* spp. and *Toxoplasma* spp., so the association with foamy macrophages might not be leptospirosis-specific (*29*). However, *Leptospira* infection in hamsters, and now deer mice, is to our knowledge the only model to produce foamy macrophages in circulation. A small portion of foamy macrophages were produced in a minority of deer mice injected with HAN media absent of leptospires. Although the mechanism behind foamy macrophage formation remains unknown in the context of leptospirosis, HAN media is rich in fatty acids and nutrients (*22*), and some level of immune activation from the intraperitoneal challenge could conceivably have resulted in rare circulating foamy macrophages.

In cases of human leptospirosis, primary kidney lesions of acute tubular necrosis and acute interstitial nephritis are observed, which were also seen in deer mice histology. Both LR131- and LAD1-challenged deer mice had acute nephritis, primarily affecting the renal tubules and with more severe lesions in LR131-challenged tissues. Renal failure might have resulted in the LR131-infected animals reaching severe endpoints before their LAD1-infected counterparts. On histologic sections, suspected foamy macrophages in challenged deer mice were only associated with renal omentum. When conducting hematoxylin and eosin staining on deer mice samples in this study, identifying foamy macrophages was more complex than in hamsters because the clear round vacuoles associated with lipid content were also associated with smaller foamy diffuse vacuoles and, sometimes, erythrophagocytosis. Histologic findings of foamy macrophages, macrophages with cytoplasmic vacuoles, and the erythrophagocytosis in either macrophages or foamy macrophage tended to overlap. Collectively, this finding suggests that foamy macrophages might phagocytose erythrocytes (*30*).

Human leptospirosis patients who experience Weil’s syndrome have a high mortality rate (≈10%) because of renal and hepatic failure (*31*) associated with direct damage of hepatocytes and Kupffer cells (*32*,*33*). LAD1-challenged deer mice had lesions that ranged from mild hepatocyte swelling (cell edema) with vacuolar hepatopathy, often in a centrilobular pattern, to severe necrotizing hepatitis with a midzonal to centrilobular pattern. The milder lesions of hepatocyte vacuolar change and cell swelling are not specific and appeared more consistent with the foamy cytoplasm of hydropic degeneration that can result from hypoxia, anorexia, or altered metabolic or nutritional states. Alternatively, those lesions might precede the more advanced necrotizing hepatitis, considering clinical signs ranged from absent to mild in the LAD1-challenged deer mice with milder hepatic lesions.

Bacterial burden data illustrate high pathogen loads in major organs of interest; the highest burden of leptospires per gram was found in liver tissue, not kidney tissue (Figure 9). That finding is consistent with pathogenesis tracking of acutely ill hamsters challenged with *L. interrogans*, where heavy dissemination to immune responsive organs is apparent (*34*). Over time in nonfatal conditions, host immune responses control leptospires in most systems, whereas persistent colonization of the kidneys enables persistent shedding. Most notable differences in burden data are reflected in the significantly reduced bacterial load in female LAD1-challenged animals and female controls (Figure 9). That finding supports the sex effect differences reported but also suggests that reduced disease severity in those animals is associated with reduced leptospire presence in liver and kidneys. Although unsurprising, the same was not true of LR131-challenged female mice and female controls, suggesting *L. interrogans* and *L. borgpetersenii* pathogenesis and interactions with host sex could be different. Although a *Leptospira* dose response was not evaluated in this study, follow-up research with the concurrent investigation of alternative routes of inoculum administration, such as conjunctival challenge, could be warranted.

Sex effects are associated with leptospirosis in humans and animals, including hamsters, and consistently favor more severe disease in male infectees (*35*–*37*). We found the same; male deer mice succumbed more quickly to severe disease endpoints (Figure 1), produced more circulating foamy macrophages (Figure 5), and harbored higher bacterial burdens in kidney and liver samples (Figure 9). Of note, *Borrelia* infection studies in *P. leucopus* mice show no difference between male and female littermates (*28*), but other work illustrates older male deer mice caught in the wild were more likely to harbor tick infestation (*38*). The mechanism of the leptospirosis sex effect is still unknown, but work with hamsters in leishmaniasis showed that treating female hamsters with testosterone increased the size of lesions (*39*), suggesting androgens might play a role in pathogenesis. In other rodent models of bacterial disease with sex effects, sex differences of *Listeria monocytogenes* in mice (more resistance in males) found that more severe disease was associated with increased levels of interleukin-10 (IL-10), and IL-10 knockout mice showed no differences between sexes (*40*). Of additional relevance, M2 macrophages can secrete IL-10, illustrating another immune component that should be investigated in the deer mouse model of leptospirosis.

In conclusion, our results demonstrate that deer mice are susceptible to acute leptospirosis. Ultimately, an alternative rodent model for acute disease is valuable. Unlike hamsters, deer mice are found readily in the wild; additional field work should survey leptospirosis prevalence among those populations. The role of circulating foamy macrophages in the pathogenesis of leptospirosis is yet to be elucidated but currently appears limited to the Cricetidae family. Collectively, this work demonstrates that deer mice represent a possible real-world host that could enable further modeling of transmission kinetics and pathogenesis of leptospirosis.

Appendix Additional information about *Peromyscus* spp. deer mice as rodent model of acute leptospirosis
