# Supplementary material for: Peromyscus spp. Deer Mice as Rodent Model of Acute Leptospirosis
Source: Emerg Infect Dis. 2025 Jul;31(7):1365–76. doi: 10.3201/eid3107.241579 (PMC12205440; doi:10.3201/eid3107.241579)
Supplement: Appendix — Additional information about Peromyscus spp. deer mice as rodent model of acute leptospirosis [file 24-1579-Techapp-s1.pdf]

Article DOI: https://doi.org/10.3201/eid3107.241579

EID cannot ensure accessibility for supplementary materials supplied by authors. Readers who have difficulty accessing supplementary content should contact the authors for assistance.

Peromyscus spp. Deer Mice as Rodent Model of Acute Leptospirosis

Appendix

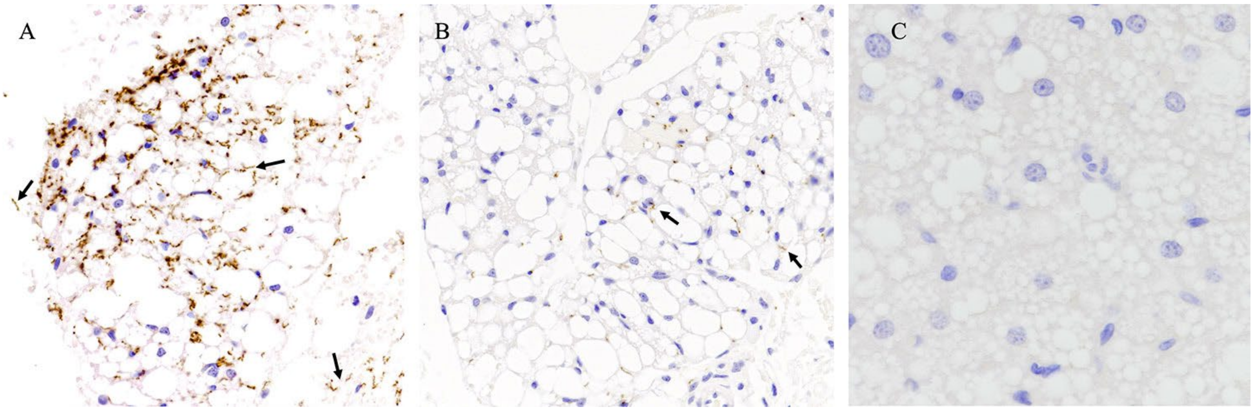

Appendix Figure. Immunohistochemistry of kidney associated adipose tissue. Shown are LipL32 reactive staining from renal adipose tissue from deermice infected with *Leptospira borgpetersenii* strain LR131 (A) and *Leptospira interrogans* strain LAD1 (B). Black arrows indicate individual leptospires. 40x original magnification. (C) Depicts adipose tissue from a non-infected individual, original magnification 40x.
